# Community stressors (violence, victimization, and neighborhood disorder) with cardiometabolic outcomes in urban Jamaica

**DOI:** 10.3389/fpubh.2023.1130830

**Published:** 2023-06-06

**Authors:** Tiffany L. Gary-Webb, Harika Dyer, Joette Mckenzie, Novie Younger-Coleman, Marshall Tulloch-Reid, Alphanso Blake, Ishtar Govia, Nadia Bennett, Shelly McFarlane, Rainford J. Wilks, David R. Williams, Trevor S. Ferguson

**Affiliations:** ^1^Department of Epidemiology, School of Public Health, University of Pittsburgh, Pittsburgh, PA, United States; ^2^Caribbean Institute for Health Research (CAIHR), The University of the West Indies, Mona, Kingston, Jamaica; ^3^Department of Social and Behavioral Sciences, Harvard T. H. Chan School of Public Health, Boston, MA, United States

**Keywords:** cardiometabolic risk, Black, community, stressors, Caribbean (Jamaica)

## Abstract

**Background:**

Despite limited data on neighborhood factors and health risk in Caribbean populations, previous analyses from Jamaica have shown that neighborhood and home disorder were associated with lower physical activity and higher cumulative biological risk among women, while poorer neighborhood infrastructure was associated with higher overweight/obesity among men.

**Design:**

Cross-sectional survey design.

**Objectives:**

In this study, we explored whether community stressors, as measured by community violence, victimization and neighborhood disorder scores, were associated with cardiometabolic outcomes (obesity, diabetes, hypertension and high cholesterol) in urban Jamaican communities. Sex-specific Poisson regression models were used to estimate prevalence ratios (PR) for these associations, adjusting for age, education, diet, physical activity and smoking.

**Participants:**

Of the 849 participants (M = 282; *F* = 567), mean age was 48 ± 18.5 years and most had at least a high school education. Men were more likely to be current smokers (29.4 vs. 10.6%) and adequately physically active (53.2 vs. 42.0%); more women were obese (46.0 vs. 19.0%), more likely to have hypertension (52.9 vs. 45.4%) and had high cholesterol (34.2 vs. 21.6%) (all *p* < 0.05).

**Results:**

We observed significant associations only for those in the middle tertile of neighborhood disorder with prevalence of higher cholesterol [PR:1.72 (1.20 to 2.47)] in women and lower prevalence of obesity [PR:0.24 (0.10 to 0.53)] in men.

**Conclusion:**

Results suggest that higher, but not the highest level of neighborhood disorder was associated with higher cholesterol levels in women and lower obesity in men. Future work will explore additional approaches to measuring neighborhood characteristics in Jamaica and the mechanisms that may underlie any relationships that are identified.

## Introduction

While there are limited data on health disparities in cardiometabolic health in Caribbean populations, recent studies on disparities among Black Caribbean populations found that there is a higher prevalence of hypertension and diabetes among Caribbean Black populations compared to West African Black populations and European white populations as well as for those with lower socioeconomic status (SES), but lower prevalence of diabetes and hypertension when compared to African Americans (1, 2). Additionally, analyses using survey data from Jamaica have shown that the prevalence of diabetes was higher among men with less education and rates of hypertension, obesity and hypercholesterolemia were higher among less educated younger women (1–3). Individual-level socioeconomic factors alone do not explain the risk and there is increasing evidence that the social determinants of health including, economic stability, neighborhood and built environment, education access and quality, healthcare access and quality and social and community context, are major contributors to disease (4, 5).

Extensive data from the U.S. show that potential contributors to poor cardiometabolic health are neighborhood physical (housing, built environment, and environmental exposures) and social (community and society context, social cohesion, and social capital) environments specifically (6–8). However, only a few studies using Jamaican data have examined the neighborhood context and none have examined perceived community stressors. A recent study using data from a sample of residents in urban Jamaica evaluated SES determined by educational attainment, household assets and community property values with ideal cardiovascular health (9). Results showed that among men, those living in communities with lower median land values (MLV), property values as a proxy for neighborhood level socioeconomic status, had lower odds of ideal cardiovascular health compared to those living in communities with high MLV. The opposite was shown in women. Likewise, in older data (2008), poorer neighborhood infrastructure (as measured by distance to open space or public parks for physical activity) was associated with higher rates of overweight/obesity among men (10). In the US context, there is some evidence that community stressors can be associated with worse cardiometabolic outcomes (11). However, only one study in the Jamaican context evaluated interviewer perceptions of neighborhood disorder and home disorder, which were associated with lower levels of physical activity and more cumulative biological risk among women (12). This manuscript is the first study to explore associations of perceptions of community stressors as measured by community violence, victimization, and neighborhood disorder, with cardiometabolic outcomes (obesity, diabetes, hypertension, and high cholesterol) in urban Jamaica. We hypothesized that more community stressors would be associated with poorer cardiometabolic health.

## Methods

### Study sample

We conducted a cross-sectional analysis using data from 849 participants in urban Jamaica. Details of the study design have been previously published (3, 9). Briefly, the sample included non-institutionalized males and females, 15 years and older, selected from four parishes in the Jamaica southeast health region, Kington, St Andrew, St Catherine, and St Thomas. These four parishes include 47% of the national population^1^ and includes all socioeconomic strata. Sampling was based on the previously conducted national survey the Jamaica Health and Lifestyle Survey 2016–2017 (3, 9), which used a multi-stage sampling procedure to recruit a nationally representative sample; response rate was 75%. Primary sampling units were randomly selected enumerations districts selected from each parish with probability proportionate to size, so that the number of selected enumeration districts were proportional to the population of the parish. Within each enumeration district, 20 households were selected using systematic sampling from a random starting point. Within each household one participant was selected using the Kish method (13). For the present study we included 44 urban enumeration districts from the four parishes mentioned above. Urban designation was based on classification of enumeration districts by the Statistical Institute of Jamaica, Geographic Services Unit.^2^ Enumeration districts were the smallest geographical district used in the national household census and comprised approximately 150 dwellings in urban areas (14). Community in this study was defined using electoral districts. Participants provided written informed consent and the study was approved by the University of the West Indies (UWI) Mona Campus Research Ethics Committee (MCREC) (ECP89 16/17). Patients or the public were not involved in the design, conduct, reporting, or dissemination plans of our research. For participants under 18 years, parents gave informed consent and the participants gave assent. All participants were given a report of their results, with explanation, as feedback.

### Study instruments and measured outcomes

Trained interviewers collected data on socio-demographic factors, community stressors, and performed anthropometric and blood pressure measurements. Data on community stressors were based on an instrument from the Chicago Community Health Study as referenced by Sternthal (15, 16). The community stressors instrument measures: 1) community violence, with questions related to fighting, robbery or mugging, 2) victimization, with questions related to being a victim of violence, robbery or vandalism and 3) disorder, with questions related to personal assault, property damage and instances of graffiti on buildings and unsupervised children (*α* = 0.80, statistic for reliability). The community stressors item responses were summed, yielding for community violence (range 5–20) and disorder (range 5–20) scores which were each categorized into sex-specific “lower,” “middle” and “upper” tertiles to represent low, moderate and high scores (higher scores indicated worse stressors). Responses concerning victimization events were summed (range 0–4) and classified as: 0 events, 1 event, and 2+ events. Measurement of blood pressure (BP) and anthropometry (height and weight) followed standardized procedures (4), and cholesterol and glucose were obtained from a finger prick blood sample. Obesity was defined as BMI ≥ 30 kg/m^2^ (17). Prior work by this team found that BMI in Jamaican 15–19 years old showed that prevalence estimated using internal Z-scores was similar to that using the International Obesity Taskforce cut-off points, thus we did not use age specific cut-off points for those 15–18 (18). Hypertension was defined as BP ≥ 140/90 mmHg or doctor diagnosed hypertension; diabetes was defined as fasting glucose ≥7.0 mmol/L or doctor diagnosed diabetes; high cholesterol was defined as total cholesterol ≥5.2 mmol/L or doctor diagnosed high cholesterol. Cardiometabolic outcomes were defined using these clinically relevant categories based on World Health Organization (19, 20) and ATP-III criteria (21).

### Data analysis

We conducted statistical analyses using Stata version 17.0. Descriptive analysis quantified the distributions of the quantitative variables including those regarded as potential confounders. Crude and sex-stratified associations were assessed using t-tests and χ^2^ tests as appropriate. Inverse probability weighting and post-stratification were applied to account for survey sampling design and the age and sex distribution of the Jamaican population. Base sampling weights were determined using the inverse of the probability of selection of each enumeration district from a parish and the inverse of the probability of selection of each household from each enumeration district. Post-stratification based on the parish by 5-year-age by sex distribution of the urban population 15 years and older in the included parishes calibrated the base weights. Estimates from weighted analyses are therefore considered representative of the urban population of Jamaicans 15 years and older in the included parishes and, by extension, urban Jamaica.

Sex-specific weighted Poisson regression models were used to estimate prevalence ratios (PR) for each outcome adjusting for age, education level, physical activity, diet, and current smoking as covariates. Interaction terms were added to the models to determine whether sex modified the associations given differences in socioeconomic status and cardiometabolic outcomes for Jamaican men and women in prior work. A list-wise deletion approach was applied to missing data. Of 849 participants in the dataset, 85% (719) had no missing values for the 12 variables included in the analysis, while 15% (130) had one or more missing values. Community violence had the most missing data among exposure variables at 9% (75). Obesity had the most missing data among outcome variables at 4% (35). Data was missing for <1% of participants for all other exposure and outcome variables. Differences between participants with missing data and those with non-missing data were not explored.

## Results

Sociodemographic, community stressors and cardiometabolic characteristics of the study population are located in Table 1. *p*-values associated with t-tests for continuous variables and χ^2^ tests for categorical variables were also reported. Of the 849 participants (*M* = 282; *F* = 567), mean age was 48 ± 18.5 years and most had at least a high school education (77.7%). Men were more likely to be current smokers (29.4 vs. 10.6%) and adequately physically active (53.2 vs. 42.0%). Men reported significantly higher scores on the community violence scale and women reported significantly higher scores on the neighborhood disorder scale. More women were obese (46.0 vs. 19.0%), more likely to have hypertension (52.9 vs. 45.4%) and have high cholesterol (34.2 vs. 21.6%) (all *χ*^2^ *p* < 0.05).

**Table 1:** Sociodemographic, community stressors, cardiometabolic characteristics of the study population stratified by sex.

	Women (*n* = 567)	Men (*n* = 282)	Total (*n* = 849)	*p*-value
	**Mean** (*SD*)	**Mean** (*SD*)	**Mean** (*SD*)	
Age (years)	48.2 (18.0)	46.6 (19.3)	47.7 (18.5)	0.235
	***n* (%)**	***n* (%)**	***n* (%)**	
**Education category**				0.984
Less than high school	125 (22.4)	62 (22.1)	187 (22.3)	
High school	291 (52.2)	146 (52.0)	437 (52.1)	
More than high school	142 (25.4)	73 (26.0)	215 (25.6)	
Current smoking	60 (10.6)	83 (29.4)	143 (16.8)	<0.001
Healthy diet (≥3 of 4 dietary factors)^a^	137 (24.2)	63 (22.3)	200 (23.6)	0.556
Adequate physical activity (≥150 min of moderate or 75 min of vigorous activity per week)^b^	236 (42.0)	148 (53.2)	384 (45.7)	0.002
	***n* (%)**	***n* (%)**	***n* (%)**	
**Community violence score (5–20)**^c^				0.043
Lower tertile	183 (35.5)	93 (35.9)	276 (35.7)	
Middle tertile	207 (40.2)	84 (32.4)	291 (37.6)	
Upper tertile	125 (24.3)	82 (31.7)	207 (26.7)	
**Victimization score (0–4)**				0.114
No victimization (0)	397 (70.1)	177 (63.4)	574 (67.9)	
Low victimization (1)	108 (19.1)	61 (21.9)	169 (20.0)	
High victimization (≥2)	61 (10.8)	41 (14.7)	102 (12.1)	
**Neighborhood disorder score (5–20)**^d^				0.015
Lower tertile	189 (33.7)	100 (35.7)	289 (34.4)	
Middle tertile	192 (34.2)	116 (41.4)	308 (36.6)	
Upper tertile	180 (32.1)	64 (22.9)	244 (29.0)	
Obese (BMI ≥30)	251 (46.0)	51 (19.0)	302 (37.1)	<0.001
Measured or self-reported doctor diagnosis of diabetes	101 (17.8)	41 (14.5)	142 (16.7)	0.225
Measured or self-reported doctor diagnosis of hypertension	300 (52.9)	128 (45.4)	428 (50.4)	0.039
Measured or self-reported doctor diagnosis of high cholesterol	194 (34.2)	61 (21.6)	255 (30.0)	<0.001

a Participants were classified as having a healthy diet if they had three or more of the following dietary characteristics: (1) low-salt diet, defined as no added salt at the table and rarely or never eats processed foods; (2) low sugar-sweetened beverage (SSB) consumption, defined as SSB intake less than twice per week; (3) adequate fruit and vegetable consumption, defined as intake of fruits or vegetables three or more times per day; and (4) adequate fish consumption, defined as eating fish two or more times per week (9).

b Participants were classified as having adequate physical activity if they engaged in at least 150 min of moderate physical activity or 75 min of vigorous activity within the past 7 days. Moderate activity was defined as taking moderate physical effort, causing the participant to breathe somewhat harder than normal. Vigorous activity was defined as taking extra physical effort, causing the participant to breathe much harder than normal. Participants were asked to consider only physical activities that they engaged in for at least 10 min at a time. For physical activity we used definitions of Ideal health that incorporated ages 12–19 (26).

c Tertiles of community violence, female: lower (5, 6), middle (7–11), upper (12–20). Male: lower (5, 6), middle (7–10), upper (11–20).

d Tertiles of neighborhood disorder, female: lower (5–9), middle (10–13), upper (14–20). Male: lower (5–10), middle (11–14), upper (15–20).

Table 2 shows the adjusted prevalence ratios for community stressors with each cardiometabolic outcome for women. The middle tertile of community violence (PR: 1.65) was marginally associated with more diabetes (*p* = 0.058). The middle tertile of neighborhood disorder was significantly associated with high cholesterol [PR:1.72, 95% CI (1.20 to 2.47)] compared to the lowest tertile.

**Table 2:** Prevalence ratio (PR) for the association between community stressors and cardiometabolic outcomes among women in urban Jamaica.

Characteristics	Obesity		Diabetes		Hypertension		High Cholesterol	
	PR (95% CI)	*p*-value	PR (95% CI)	*p*-value	PR (95% CI)	*p*-value	PR (95% CI)	*p*-value
**Community violence score**								
Lower tertile (score: 5–6)	ref	–	ref	–	ref	–	ref	–
Middle tertile (score: 7–11)	1.04 (0.78 to 1.39)	0.766	1.65 (0.98 to 2.78)	0.058	1.17 (0.78 to 1.73)	0.428	1.10 (0.76 to 1.61)	0.599
Upper tertile (score: 12–20)	1.22 (0.90 to 1.66)	0.195	1.48 (0.70 to 3.14)	0.298	0.90 (0.59 to 1.38)	0.629	0.74 (0.43 to 1.27)	0.266
**Victimization score**								
No victimization (score: 0)	ref	–	ref	–	ref	–	ref	–
Low victimization (score: 1)	1.02 (0.74 to 1.41)	0.911	1.29 (0.66 to 2.50)	0.449	1.17 (0.86 to 1.61)	0.308	1.19 (0.88 to 1.63)	0.251
High victimization (score: ≥2)	0.69 (0.35 to 1.35)	0.270	0.82 (0.34 to 1.97)	0.653	0.80 (0.46 to 1.37)	0.406	1.33 (0.72 to 2.47)	0.350
**Neighborhood disorder score**								
Lower tertile (score: 5–9)	ref	–	ref	–	ref	–	ref	–
Middle tertile (score: 10–13)	0.86 (0.62 to 1.19)	0.358	1.34 (0.74 to 2.43)	0.327	1.14 (0.84 to 1.56)	0.395	1.72 (1.20 to 2.47)	0.004
Upper tertile (score: 14–20)	1.01 (0.71 to 1.43)	0.969	1.31 (0.69 to 2.51)	0.402	1.28 (0.92 to 1.78)	0.133	0.92 (0.56 to 1.51)	0.732

Adjusted for age, education category, current smoking, healthy diet, and adequate physical activity.

For men (Table 3), those in the middle tertile of neighborhood disorder had a significantly lower prevalence of obesity [PR:0.24, 95% CI (0.10 to 0.53)] compared to those in the lowest tertile. There was no significant association for those in the highest tertile of neighborhood disorder compared to the lowest tertile. Several other associations reached marginal significance (*p* > 0.05, *p* < 0.10). The highest tertile of community violence (PR: 0.55) was marginally associated with a lower prevalence of obesity, and the middle tertile of community violence (PR:1.51) was marginally associated with a higher prevalence of hypertension. Experiencing one event of victimization (PR: 2.20) was marginally associated with higher prevalence of diabetes. The middle tertile of neighborhood disorder (PR: 0.54) was marginally associated with lower prevalence of high cholesterol.

**Table 3:** Prevalence ratio (PR) for the association between community stressors and cardiometabolic outcomes among men in urban Jamaica.

Characteristics	Obesity		Diabetes		Hypertension		High Cholesterol	
	PR (95% CI)	*p*-value	PR (95% CI)	*p*-value	PR (95% CI)	*p*-value	PR (95% CI)	*p*-value
**Community violence score**								
Lower tertile (score: 5–6)	ref	–	ref	–	ref	–	ref	–
Middle tertile (score: 7–10)	0.60 (0.32 to 1.13)	0.108	1.15 (0.49 to 2.71)	0.747	1.51 (0.96 to 2.37)	0.071	1.89 (0.81 to 4.42)	0.137
Upper tertile (score: 11–20)	0.55 (0.30 to 1.02)	0.056	0.80 (0.31 to 2.10)	0.647	0.80 (0.52 to 1.24)	0.307	0.70 (0.22 to 2.17)	0.525
**Victimization score**								
No victimization (score: 0)	ref	–	ref	–	ref	–	ref	–
Low victimization (score: 1)	0.77 (0.44 to 1.33)	0.339	2.20 (0.91 to 5.33)	0.079	1.07 (0.65 to 1.78)	0.780	1.15 (0.48 to 2.76)	0.751
High victimization (score: ≥2)	1.09 (0.56 to 2.14)	0.788	0.83 (0.18 to 3.89)	0.812	0.77 (0.47 to 1.25)	0.280	1.02 (0.38 to 2.75)	0.969
**Neighborhood disorder score**								
Lower tertile (score: 5–10)	ref	–	ref	–	ref	–	ref	–
Middle tertile (score: 11–14)	0.24 (0.10 to 0.53)	0.001	0.70 (0.35 to 1.42)	0.312	0.95 (0.66 to 1.37)	0.776	0.54 (0.27 to 1.08)	0.079
Upper tertile (score: 15–20)	0.51 (0.18 to 1.47)	0.208	0.69 (0.29 to 1.67)	0.405	0.79 (0.49 to 1.27)	0.318	0.40 (0.12 to 1.32)	0.129

Adjusted for age, education category, current smoking, healthy diet, and adequate physical activity.

Sensitivity analyses were performed for participants who had high measured values for diabetes, hypertension, and high cholesterol, but did not have a doctor’s diagnosis and were labeled as undiagnosed. Cross tabulations with *χ*^2^ tests comparing socio demographics and community stressors for undiagnosed participants with those who reported doctor diagnosis of the outcomes showed that the undiagnosed tended to be younger and included more current smokers (data not shown). Although the interaction terms between community stressors and sex were statistically significant for the obesity outcome only, analyses were stratified by sex for all outcomes.

## Discussion

To our knowledge, this is the first study to examine association of perceptions of community stressors with measured cardiometabolic outcomes in a robust sample of participants from urban Jamaica. While we hypothesized that higher scores of community stressors would show a linear association with poorer cardiometabolic health, we observed significant associations only for those in the middle tertile of neighborhood disorder with prevalence of higher cholesterol in women and lower prevalence of obesity in men.

Limited data on community-level stressors are available for comparison purposes in Jamaica or the Caribbean. Analyses using the Jamaican Health and Lifestyle Survey II (2008) have shown that more neighborhood disorder, as measured by interviewer perceptions of the conditions of home, streets and yards, amount of noise and air quality of neighborhoods, was associated with poorer cumulative biological risk (a combined marker of cardiometabolic health) among women (12). Further, in the same dataset, increased mean waist circumference (measure of central obesity) was higher for those living in areas with increased crime (22). Gender differences have been shown in prior studies conducted by the authors and have been different across several outcomes. In this analysis, men reported significantly higher levels of neighborhood disorder in the middle tertile than women, but interestingly not in the highest tertile. While these differences are not fully understood, our group has been stratifying data by gender and implementing sex-specific models to assure that we are documenting and actively exploring these relationships.

Our finding that the middle tertile of neighborhood disorder was associated with cholesterol and obesity and not the highest tertile did not support our hypothesis that this relationship would be linear. This finding is not unique to data from Jamaica, however, there are several other relationships that have shown a non-linear relationship in Jamaican datasets, including income with blood pressure (23) and obesity (22, 24), indicating higher levels for both high and low incomes. Given that these data are cross-sectional, causal inferences are not appropriate. However, it should be noted that Jamaica is going through an epidemiologic transition where there is a co-existence of obesity with undernutrition as is the case in many Low- and Middle-Income Countries (LMIC) (22). These changing population patterns indicate that the patterns may be shifting to the low socioeconomic status and worse chronic disease pattern that we see in US populations (4). Another possible explanation could be how residents experience stress at the neighborhood and individual level. There are no data to understand whether there were homogenous experiences of stressors, however, exploring this concept in a future study is warranted.

Despite its strengths, the analysis has several limitations including limited measures of socio-economic status as income is chronically underreported. Another limitation was the missing data (9%) for the community violence scale and finally, the study has no measure of alcohol intake which could serve as a potential confounder in our analyses (25). These analyses will contribute to the literature on neighborhood and health in the Jamaican context and allow for other studies to build on these measures which incorporate participant perceptions of their neighborhood.

## Conclusion

Results suggest that higher, but not the highest level of neighborhood disorder was associated with higher cholesterol levels in women and lower obesity in men. Future work will explore additional approaches to measuring neighborhood characteristics in Jamaica and the mechanisms that may underlie any relationships that are identified. Future research from our team will explore novel measures of neighborhood-level SES (property sales and land values) and the relationship to other (individual and neighborhood-level) socioeconomic variables and with cardiometabolic outcomes. We also plan to further explore mechanisms contributing to neighborhoods and health in Jamaican datasets, and eventually compare these to cohort data for Black Americans.

## Data availability statement

The raw data supporting the conclusions of this article will be made available by the authors, without undue reservation.

## Ethics statement

The studies involving human participants were reviewed and approved by the University of the West Indies (UWI) Mona Campus Research Ethics Committee (MCREC) (ECP89 16/17). The patients/participants provided their written informed consent to participate in this study.

## Author contributions

TG-W and TF conceptualized and led the analysis and drafting of the manuscript. TF conducted the original survey along with many of the authors who are co-investigators. HD conducted statistical analysis under the advisement of TG-W, TF, and NY-C. All authors contributed to the article and approved the submitted version.

## Funding

This research was supported by the Bernard Lown Scholars in Cardiovascular Health Program of the Department of Global Health and Population of the Harvard T.H. Chan School of Public Health (grant #: BLSCHP-1604). This work was also supported by a ‘Year of Global’ internal grant from the University of Pittsburgh.

## Conflict of interest

The authors declare that the research was conducted in the absence of any commercial or financial relationships that could be construed as a potential conflict of interest.

## Publisher’s note

All claims expressed in this article are solely those of the authors and do not necessarily represent those of their affiliated organizations, or those of the publisher, the editors and the reviewers. Any product that may be evaluated in this article, or claim that may be made by its manufacturer, is not guaranteed or endorsed by the publisher.

## Footnotes

^1^ https://statinja.gov.jm/Demo_SocialStats/populationbyparish.aspx

^2^ https://statinja.gov.jm/maps.aspx
